# Clinicopathologic Predictors of Survival Following Oral Cancer Surgery: A Retrospective Cohort Study

**DOI:** 10.3390/cancers17152454

**Published:** 2025-07-24

**Authors:** Katarzyna Stawarz, Karolina Bieńkowska-Pluta, Adam Galazka, Anna Gorzelnik, Monika Durzynska, Magdalena Misiak-Galazka, Grzegorz Stawarz, Jakub Zwolinski

**Affiliations:** 1 Head and Neck Cancer Department, Maria Sklodowska-Curie National Research Institute of Oncology, 02-781 Warsaw, Poland; 2 Department of Pathology, Maria Sklodowska-Curie National Research Institute of Oncology, 02-781 Warsaw, Poland; 3 Department of Urology and Urological Oncology, Multidisciplinary Hospital in Warsaw-Miedzylesie, Bursztynowa St. 2, 04-479 Warsaw, Poland

**Keywords:** oral squamous cell carcinoma, perineural invasion, angioinvasion, lymphatic invasion, tumor volume, disease-specific survival

## Abstract

**Background**: Despite advances in treatment, oral squamous cell carcinoma (OSCC) remains associated with high recurrence and mortality rates. Traditional TNM staging, while foundational, may not fully capture tumor aggressiveness. This study aimed to identify clinical and histopathological predictors of survival to enhance risk stratification and guide treatment planning in OSCC patients. **Methods**: A retrospective study of 100 patients with confirmed OSCC treated surgically with curative intent between January 2019 and January 2024 was analyzed. Clinicopathologic variables—including tumor volume, angioinvasion, perineural invasion, lymphatic invasion, and nodal status—were evaluated. Disease-specific survival (DSS) was assessed using Kaplan–Meier estimates, Cox regression, and logistic regression models. **Results**: The cohort had a mean age of 62.1 years, with a 46% OS rate and 43% DSS at study end. Perineural invasion (44%) and lymphatic invasion (42%) were the most common invasive features. Kaplan–Meier analysis revealed significantly reduced DSS in patients with angioinvasion, perineural invasion, and pN+ status. Multivariate logistic regression identified perineural invasion (OR = 3.93, *p* = 0.0023) and pN+ status (OR = 2.74, *p* = 0.0284) as independent predictors of cancer-specific mortality. Tumor volume was significantly associated with lymphatic invasion but not directly with DSS. **Conclusions**: Perineural invasion, angioinvasion, lymph node involvement, and tumor volume are important prognostic markers in OSCC, offering critical information beyond TNM staging. Incorporating these features into risk assessment models could improve prognostic accuracy and inform more individualized treatment strategies for high-risk OSCC patients.

## Simple Summary

Oral squamous cell carcinoma (OSCC) continues to carry high rates of recurrence and mortality, despite advancements in treatment. This retrospective study analyzed 100 surgically treated OSCC patients to identify clinical and histopathological predictors of survival that go beyond traditional TNM staging. Key variables assessed included tumor volume, angioinvasion, perineural invasion, lymphatic invasion, and nodal involvement. The average patient age was 62.1 years, with a 46% overall survival and 43% disease-specific survival (DSS) at the study’s conclusion. Perineural and lymphatic invasion were the most prevalent invasive features. Kaplan–Meier analysis showed that angioinvasion, perineural invasion, and pN+ nodal status significantly reduced DSS. Multivariate analysis confirmed perineural invasion and pN+ status as independent predictors of cancer-specific mortality. Tumor volume was associated with lymphatic invasion but not directly with DSS. The study concludes that integrating these histopathologic markers into current staging systems could enhance prognostic precision and support more personalized treatment planning for high-risk OSCC patients.

## 1. Introduction

Despite advances in treatment, the global burden of oral squamous cell carcinoma (OSCC) remains substantial, with approximately 377,000 new cases diagnosed annually and an estimated 177,000 deaths reported worldwide in 2020. Incidence and mortality rates continue to rise in many regions, largely driven by persistent exposure to preventable risk factors such as tobacco use, alcohol consumption, and limited access to early detection and care [1,2]. The disease disproportionately affects populations with high prevalence of tobacco use, alcohol consumption, and poor oral hygiene. According to the IARC (International Agency for Research on Cancer) Handbooks of Cancer Prevention, primary prevention strategies such as tobacco cessation, harmful alcohol use reduction, early detection, and visual screening programs are essential in reducing OSCC incidence and improving survival outcomes [3]. Despite advances in treatment modalities, survival remains suboptimal, particularly in low- and middle-income countries where access to timely diagnosis and care is limited.

While early-stage OSCC is typically managed with surgery alone, more advanced stages often necessitate adjuvant therapies such as radiotherapy (RTH) or chemoradiotherapy (CHTH-RTH) [3]. Nevertheless, even with aggressive multimodal treatment, recurrence rates remain high, estimated at 21–47.1% [4,5,6]. Unlike oropharyngeal squamous cell carcinoma, where human papillomavirus (HPV) status has well-established prognostic relevance, the vast majority of OSCC are HPV-negative. As such, clinical outcomes in OSCC are less influenced by viral oncogenesis and more strongly determined by conventional risk factors and pathological features such as tumor stage, perineural and lymphovascular invasion, nodal involvement, and margin status. These aggressive disease characteristics often necessitate intensified treatment approaches, which may adversely affect long-term functional outcomes and quality of life [7,8]. Therefore, the identification and validation of reliable prognostic factors are crucial for guiding personalized treatment strategies and potentially reducing the risk of disease recurrence.

Although precision oncology plays a leading role in the personalized treatment of cancer patients today [9,10], there remain important clinical and histological factors that can inform treatment strategies independently of molecular profiling.

For decades, treatment planning in clinical practice has primarily been guided by the TNM staging system [11], alongside an assessment of the patient’s overall health status [12]. Although TNM remains a cornerstone of oncologic classification, it has well-recognized limitations. It provides only an anatomical overview of tumor burden and fails to incorporate tumor biology, molecular characteristics, or patient-specific factors [13,14]. As a result, individuals with identical TNM stages may exhibit markedly different clinical outcomes and therapeutic responses. Furthermore, the TNM system does not account for dynamic prognostic indicators such as treatment response, risk of recurrence, or key histopathological features, including neuroinvasion, lymphatic and vascular invasion, or tumor differentiation [15]. Consequently, TNM staging alone appears insufficient for guiding personalized, risk-adapted treatment strategies in the era of precision oncology.

To address these gaps, the identification of robust prognostic markers is essential for developing individualized treatment pathways. Such markers can improve the accuracy of outcome prediction, support informed clinical decision-making, and enable more precise risk stratification. Beyond traditional TNM staging and the consideration of patient comorbidities, integrating additional variables—such as tumor volume and histopathological invasion markers—into prognostic models may further refine risk assessment, optimize both surgical and adjuvant treatment planning, and enhance survival prediction [16]. This comprehensive and integrative approach aligns with the principles of precision medicine and holds the potential to improve both oncologic outcomes and quality of life in patients with OSCC [17].

In this study, we aimed to identify key clinical and histopathological predictors of disease-specific survival (DSS) in patients undergoing surgical treatment followed, in most cases, by adjuvant therapy for OSCC. By analyzing a comprehensive set of patient-related, tumor-related, and treatment-related variables, we sought to determine which factors are most strongly associated with long-term oncologic outcomes. The primary objective of this investigation was to improve prognostic accuracy and support the development of individualized treatment strategies, thereby enabling more effective risk stratification and guiding the selection of optimal therapeutic approaches.

## 2. Materials and Methods

### 2.1. Subjects

This retrospective cohort study included 100 patients diagnosed with primary OSCC, all of whom underwent surgical tumor resection with either unilateral or bilateral neck dissection, followed by free flap reconstruction. All surgical procedures were performed with curative intent, achieving R0 resection margins, between January 2019 and January 2024. Patients were treated at the Maria Sklodowska-Curie National Research Institute of Oncology, a tertiary referral center in Warsaw, Poland. The study included all consecutive patients meeting the inclusion criteria during the defined 5-year period, yielding a total sample of 100 patients. No formal a priori power calculation was performed, as this was an exploratory observational study based on an available clinical cohort. Ethical approval for this study was obtained from the Ethics Committee of the Maria Sklodowska-Curie National Research Institute of Oncology in Warsaw. Although no experimental interventions were conducted, ethical oversight was nonetheless secured and appropriately documented. Participants were either diagnosed within the institute or referred from other medical facilities across the country. Clinicopathological data were retrospectively extracted from medical records and histopathological reports using institutional patient and hospital identifiers. Variables with missing data were reviewed, and cases with incomplete survival or histopathological data were excluded from relevant analyses. No imputation was performed. The extent of missing data was minimal and did not affect multivariable modeling. All surgical procedures were performed under general anesthesia. The evaluated variables included patient-related factors, such as age, gender, smoking history (including pack-years), alcohol use (including alcohol-years), diabetes mellitus (DM), type of atherosclerosis, and receipt of postoperative radiotherapy (RTH) and chemotherapy (CHTH), with mean doses recorded. Tumor-related characteristics included primary tumor location, histological type, tumor grade, TNM classification, tumor volume, presence of angioinvasion, neuroinvasion, lymphatic invasion, and pathological extranodal extension (pENE).

### 2.2. Inclusion Criteria

Histopathologically confirmed diagnosis of OSCC.

Patients who underwent surgical treatment with curative intent.

Availability of complete clinical, pathological, and survival data.

Adults aged ≥18 years.

Minimum follow-up period of 12 months or until death.

### 2.3. Exclusion Criteria

Non-squamous cell carcinoma of the oral cavity.

HPV-positive oropharyngeal carcinoma.

Patients with metastatic disease at initial diagnosis.

Patients who received only palliative care or no surgical treatment.

History of prior malignancies in the head and neck region.

Lost to follow-up before meaningful outcome data could be collected.

### 2.4. Adjuvant Treatment

All patients in this study demonstrated high-risk pathological features following surgical resection, including pENE, perineural invasion, lymphovascular invasion, or advanced nodal disease (≥N2). In line with the current NCCN (National Comprehensive Cancer Network) Guidelines for Head and Neck Cancers (Version 2.2025), adjuvant treatment decisions were guided by a multidisciplinary tumor board. Postoperative radiotherapy (PORT) was administered to all patients using intensity-modulated radiation therapy (IMRT). The prescribed total dose ranged from 60 to 66 Gy, delivered in daily fractions of 1.8–2.0 Gy, five days per week. Radiation fields included the primary tumor bed and regional lymphatics, adjusted based on surgical margins and nodal burden. For patients with pENE, concurrent chemotherapy was administered with high-dose cisplatin at 100 mg/m^2^ every three weeks for up to three cycles, as recommended in the NCCN Guidelines (Version 2.2025). In cases where high-dose cisplatin was contraindicated, weekly cisplatin at 40 mg/m^2^ was used at the discretion of the treating oncologist.

### 2.5. Follow–Up Assessment

Patients were evaluated during routine clinical follow-up visits every 3 to 6 months, either for a minimum of five years or until death, with follow-up concluding on June 1, 2025. For each patient, both the date and cause of death were systematically recorded. The mean follow-up time for the cohort was 33.3 months, with a median of 42.0 months, a standard deviation of 22.7 months, and a range of 0.3 to 110.7 months. Overall survival (OS) was defined as the time from the date of primary surgery to either the last follow-up or death from any cause. Disease-specific survival (DSS) was measured from the date of surgery to death attributed specifically to the disease (death of disease, DOD). Patients who were alive at their last follow-up visit were censored at that time point.

### 2.6. Histopathological Evaluation

All surgical specimens were reviewed by experienced head and neck pathologists according to standardized protocols. Tumor volume was calculated based on gross measurements provided in pathology reports, using the ellipsoid formula when applicable. Angioinvasion, perineural invasion, and lymphatic invasion were assessed using routine hematoxylin and eosin (H&E)-stained slides. To enhance diagnostic precision, immunohistochemistry (IHC) was employed in selected cases: D2–40 was used to highlight lymphatic channels, and CD31/CD34 were used for vascular endothelium, allowing for clear differentiation between lymphatic and blood vessel invasion. Perineural invasion was defined as tumor cell infiltration into the nerve sheath or circumferential encasement of at least one-third of the nerve perimeter [18]. Immunohistochemistry (D2-40, CD31/CD34) was performed routinely in all cases diagnosed and treated in our Institute, not exclusively for this study. These markers were part of the standard diagnostic protocol for assessing lymphovascular invasion and were recorded in the histopathology reports. Resection margin status was classified according to AJCC/CAP criteria, with R0 resection defined as the absence of tumor at inked surgical margins. Regarding surgical margins, we used the >5 mm threshold to define “clear” (negative) margins, and margins between 1–4 mm were categorized as “close”. In most cases, the margins were classified as close (1–4 mm), a feature associated with increased risk of recurrence. Pathologic staging was performed using the 8th edition of the AJCC TNM classification, which incorporates the depth of invasion (DOI) in T staging and ENE in N classification. For statistical modeling, pENE was treated as an independent prognostic variable and was not used to redefine the N category, to avoid analytic redundancy.

### 2.7. Statistical Analysis

All statistical analyses were conducted using Jamovi software (version 2.6.44.0). Categorical variables were reported as frequencies and percentages, while continuous variables were summarized as means (M) with standard deviations (SDs). A stepwise selection approach was applied to construct the final model, incorporating variables based on both statistical significance and clinical relevance to avoid overfitting. Variables considered for inclusion included age, gender, smoking-years, alcohol-years, tumor location, T and N classification, angioinvasion, perineural invasion, lymphatic invasion, tumor volume, pathological extranodal extension (pENE), and receipt of postoperative RTH or CHTH. Pearson correlation coefficients were used to assess associations between continuous and categorical variables. Overall survival (OS) and disease-specific survival (DSS) were estimated using the Kaplan–Meier method (univariate survival analysis technique), and differences between survival curves were evaluated with the log-rank test. To identify prognostic factors for survival, Cox proportional hazards regression was used as the primary method of survival analysis. Variables with *p* < 0.05 in univariate Cox analysis were entered into the multivariate Cox model to adjust for potential confounding. Covariates were selected based on both clinical relevance and statistical significance to minimize model overfitting. Patients who were alive at the last follow-up were censored at that time point for both OS and DSS analyses. Additionally, logistic regression analyses were performed in an exploratory capacity to assess the association of specific pathological features with disease-specific mortality as a binary outcome. A Chi-square test was used to examine the relationship between triple invasion status (simultaneous angioinvasion, perineural invasion, and lymphatic invasion) and disease-specific mortality.

## 3. Results

### 3.1. Patient and Tumor Characteristics

**Figure 1:**
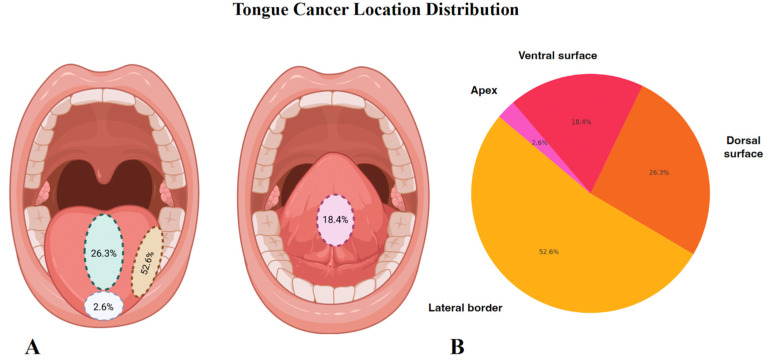
Distribution of tongue cancer locations. Anatomical distribution of tongue cancer locations among patients included in the study. (**A**) Schematic illustrations showing the percentage of tumor involvement across specific anatomical regions of the tongue, including the lateral border (52.6%), dorsal surface of the oral tongue (26.3%), ventral surface (18.4%), and apex (2.6%). (**B**) Corresponding pie chart summarizing the proportional distribution of tumor sites across the examined cohort.

A total of 100 patients were included in the study, comprising 57% (*n* = 57) male and 43% (*n* = 43) female participants. The mean age was 62.1 ± 10.3 years (M ± SD). A history of diabetes mellitus (DM) was reported in 15% of patients, while smoking and atherosclerosis were present in 60% and 40% of cases, respectively. The mean duration of tobacco use was 25.73 ± 9.65 years (mean ± SD). Alcohol consumption was confirmed in 45% of patients (*n* = 45), with a mean alcohol exposure of 8.72 ± 4.79 years. Postoperative RTH was administered in the majority of cases (64%), with a mean dose of 62.15 ± 2.90 Gy. In contrast, postoperative CHTH was administered in only 8% of patients, with a mean cumulative dose of 244.44 ± 101.38 mg/m^2^ (Table 1). The most frequent tumor location was the tongue, identified in 38% (*n* = 38) of patients, followed by the floor of the mouth (29%, *n* = 29) and the buccal mucosa (13%, *n* = 13). The lip tumors involved both the cutaneous (skin) and mucosal surfaces. Detailed data on tongue tumor distribution and other tumor locations are presented in Figure 1. Histopathological analysis confirmed squamous cell carcinoma (SCC) in all cases, with a mean tumor volume of 34.55 ± 59.38 cm^3^. Among histopathologic features, perineural invasion was the most prevalent (44%, *n* = 44), followed by lymphatic invasion (42%, *n* = 42) and angioinvasion (28%, *n* = 28). Pathologic extranodal extension (pENE) was present in 23% of cases. Regarding tumor differentiation, most tumors were graded as G2 (68%, *n* = 68), followed by G3 (24%, *n* = 24) and G1 (8%, *n* = 8). In terms of tumor staging, T3 was the most commonly reported stage (27%, *n* = 27), closely followed by T4 (26%, *n* = 26). T1 tumors were the least common, accounting for 6% (*n* = 6). Nodal involvement was observed in 19% (*n* = 19) of cases as N1, 9% (*n* = 9) as N2, and 1% (*n* = 1) as N3, while 54% (*n* = 54) of patients were staged as N0. An Eastern Cooperative Oncology Group (ECOG) performance status of 0 was observed in 33% of patients (*n* = 33), while ECOG 1 was noted in 67% (*n* = 67). A comprehensive summary of tumor-related variables is presented in Table 2 and Table 3. Pathologically confirmed nodal metastases were reported in 37% (*n* = 37). Over the course of the follow-up period, the tumor recurred in a significant number of patients—29% (*n* = 29). At the end of the complete study follow-up, almost half of the patients died of the disease—43% (*n* = 43)—while the OS rate was 46% (46). 

### 3.2. Assessment of Clinical Variables

In the correlation analysis between invasion patterns and clinical variables, angioinvasion showed a statistically significant moderate positive correlation with the administration of post-CHTH (*r* = 0.309, *p* = 0.0018). A positive trend was also observed between angioinvasion and smoking intensity, as measured in pack-years (*r* = 0.214, *p* = 0.0969), although this did not reach statistical significance. Neuroinvasion demonstrated a strong and statistically significant correlation with death from disease (*r* = 0.403, *p* < 0.0001), underscoring its adverse prognostic relevance. A positive association was also found with disease recurrence (*r* = 0.188, *p* = 0.0607), although this did not reach statistical significance. A weak negative correlation with alcohol-years was observed (*r* = −0.245, *p* = 0.1091). Lymphatic invasion was strongly and significantly associated with the total post-RTH dose (*r* = 0.684, *p* < 0.0001). A moderate but non-significant association with postoperative chemotherapy dose was noted (*r* = 0.286, *p* = 0.456), though the interpretation of this finding is limited by a small sample size (*n* = 9), (Table 4).

### 3.3. Evaluation of Certain Pathological Variables

Correlation analysis between histopathological features and invasion patterns revealed several statistically significant associations. Lymphatic invasion demonstrated a strong positive correlation with tumor volume (*r* = 0.399, *p* = 0.0002). A statistically significant negative correlation was observed between lymphatic invasion and early-stage tumors (T1) (*r* = −0.215, *p* = 0.0317), suggesting that lymphatic spread was less common in early disease. Lymphatic invasion also showed a strong positive correlation with pathological extranodal extension (pENE) (*r* = 0.519, *p* < 0.0001). Angioinvasion was positively correlated with both neuroinvasion (*r* = 0.341, *p* = 0.0015) and poorly differentiated tumors (G3) (*r* = 0.380, *p* = 0.0001). Neuroinvasion showed a moderate positive correlation with angioinvasion (*r* = 0.341, *p* = 0.0015) and a weak positive correlation with lymphatic invasion (*r* = 0.216, *p* = 0.0485), suggesting that these invasive features frequently co-occur. In addition, neuroinvasion was significantly associated with nodal metastasis (pN+) (*p* = 0.0451). No significant correlations were observed between any of the invasion types and tumor stages T2–T4 or tumor grades G1–G2, (Table 5).

### 3.4. Univariate Analysis

Univariate logistic regression analysis was performed to identify pathological predictors of disease-specific mortality. Perineural invasion emerged as a strong and statistically significant factor associated with an increased risk of death from disease (odds ratio [OR] = 5.63, *p* = 0.0001). Similarly, the presence of nodal metastasis (pN+) was significantly associated with higher disease-specific mortality (OR = 3.84, *p* = 0.0024). Other variables, including angioinvasion, lymphatic invasion, tumor volume, and pENE, showed positive but statistically non-significant associations with death from disease, (Table 6).

### 3.5. Multivariate Analysis

In the multivariate logistic regression model evaluating predictors of disease-specific mortality, both neuroinvasion and nodal metastasis (pN+) remained independently and significantly associated with an increased risk of death. Neuroinvasion demonstrated a strong association, with an odds ratio (OR) of 4.84 (95% CI: 1.97–11.85, *p* = 0.0006), indicating that patients with perineural invasion were nearly five times more likely to die from the disease compared to those without. Similarly, pN+ status was significantly associated with mortality (OR = 3.08, 95% CI: 1.22–7.80, *p* = 0.0178), confirming its role as a key prognostic factor, (Table 7).

### 3.6. Cox Regression Analysis

A multivariable Cox proportional hazard regression evaluated the impact of pN+ status and neuroinvasion on survival outcomes. In the univariate analysis, pN+ showed a non-significant trend toward increased risk of death, with a hazard ratio (HR) of 1.69 (95% CI: 0.88–3.24, *p* = 0.117). Perineural invasion was not significantly associated with survival (HR = 1.05, 95% CI: 0.54–2.08, *p* = 0.877). After adjusting for both variables in the multivariable model, neither remained significant. The HR for pN+ was 1.68 (95% CI: 0.88–3.24, *p* = 0.118), and for perineural invasion, it was 1.02 (95% CI: 0.52–2.02, *p* = 0.944). The concordance index was 0.561 and with an R-squared value of 0.045, indicating weak predictive ability. The likelihood ratio test for the model was not significant (*p* = 0.286), (Table 8).

### 3.7. Kaplan–Meyer Curves

**Figure 2:**
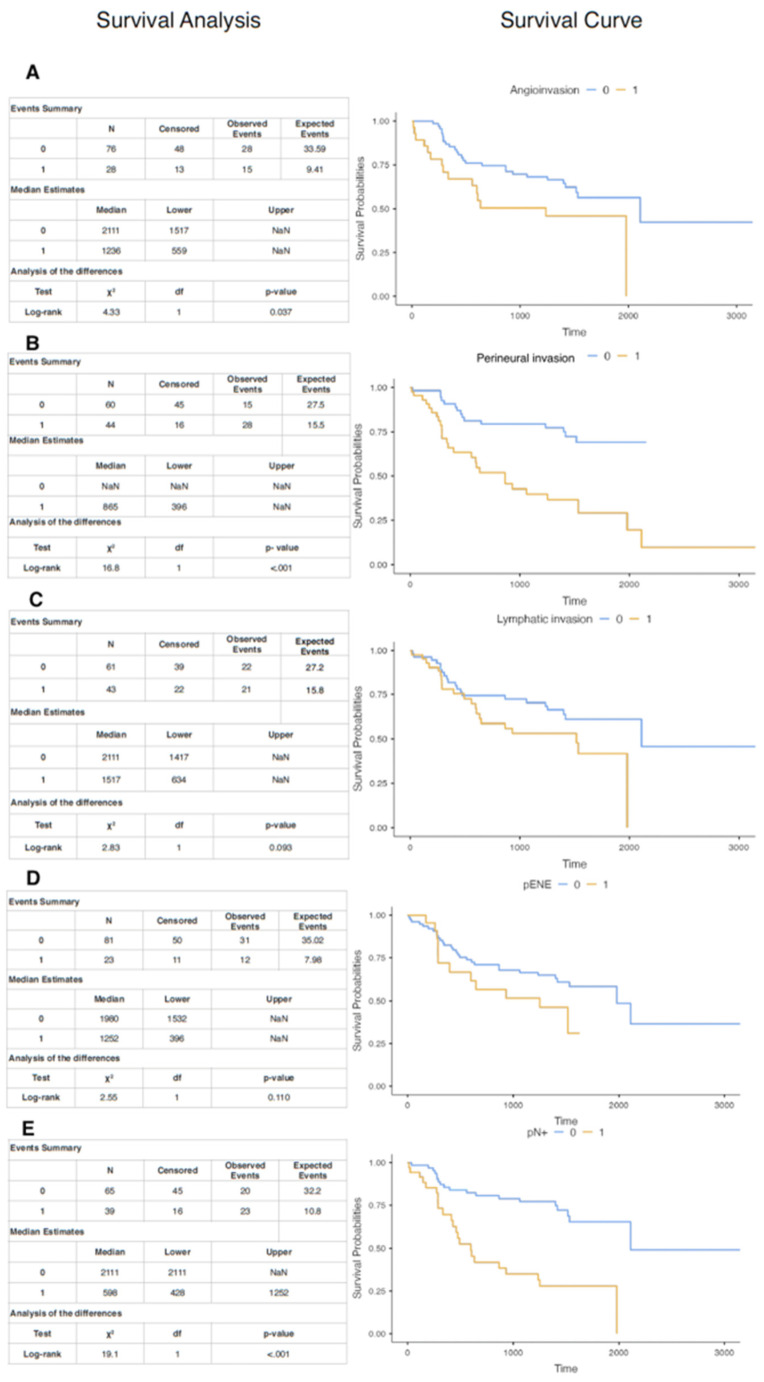
Kaplan–Meier survival curves and log-rank test results for key pathological features in OSCC patients. Panels (**A**–**E**) display survival analyses comparing patients with and without specific invasive features. (**A**) Angioinvasion was associated with significantly reduced survival (median: 1236 vs. 2111 days; *p* = 0.037). (**B**) Neuroinvasion showed a strong association with poor prognosis (median: 865 days; *p* < 0.001). (**C**) Lymphatic invasion indicated a trend toward worse survival (median: 634 vs. 2111 days), though not statistically significant (*p* = 0.093). (**D**) Pathological extranodal extension (pENE) showed a similar trend (median: 1252 vs. 1980 days; *p* = 0.110). (**E**) Positive nodal status (pN⁺) was the strongest predictor of reduced survival (median: 598 vs. 2111 days; *p* < 0.001). Survival curves are shown for patients with (1, orange) and without (0, blue) the respective features. Each panel includes summary statistics and log-rank test results.

Kaplan–Meier survival analysis demonstrated significant differences in DSS for certain pathological variables. Patients with angioinvasion experienced reduced survival compared to those without, with a median survival of 1236 days vs. 2111 days, respectively (*p* = 0.037; Panel A). Neuroinvasion was strongly associated with poorer survival (Panel B), with a shorter median survival of 865 days in patients with neuroinvasion compared to those without (*p* < 0.001). Although lymphatic invasion was associated with a shorter median survival (634 vs. 2111 days), this difference did not reach statistical significance (*p* = 0.093; Panel C). Similarly, the presence of pENE showed a trend toward reduced survival (1252 vs. 1980 days), but this difference was not statistically significant (*p* = 0.110; Panel D). In contrast, pN+ status was a strong predictor of worse survival (Panel E), with a median survival of 598 days in patients with nodal metastases vs. 2111 days in those without (*p* < 0.001), (Figure 2).

### 3.8. Combined Perineural, Vascular, and Lymphatic Invasion: Clinical Implications

A Chi-square analysis was conducted to evaluate the association between survival status and the presence of simultaneous angioinvasion, neuroinvasion, and lymphatic invasion. The results revealed a statistically significant association (χ^2^ = 5.19, df = 1, *p* = 0.023), indicating that patients with all three invasion types were significantly more likely to be deceased at follow-up compared to those without this combination. Among patients with all three invasions, 86% (12/14) were deceased, compared to 49% (42/86) in those without. Logistic regression analysis demonstrated that the presence of simultaneous angioinvasion, perineural invasion, and lymphatic invasion was significantly associated with increased odds of death. Specifically, patients exhibiting all three invasion types had an OR of 6.29 (95% CI: 1.33–29.78; *p* = 0.021), indicating that they were over six times more likely to be deceased at follow-up compared to patients without this combination of features, (Table 9).

## 4. Discussion

Oral cancer, particularly SCC, remains an aggressive malignancy with a poor prognosis despite advances in treatment strategies [19,20]. This study was restricted to primary tumors of the oral cavity, while HPV testing was performed in accordance with current diagnostic protocols and NCCN guidelines, which recommend the routine assessment of HPV status in oral cavity cancers. This approach ensured consistency in tumor classification and allowed for the appropriate exclusion of HPV-positive oropharyngeal carcinomas. SCC is most frequently diagnosed in older adults, with a reported mean age exceeding 60 years [21]. The high rate of recurrence, limited efficacy of chemotherapy, and suboptimal response to immunotherapy contribute to a 5-year OS rate estimated at approximately 68–70% [22]. These observations are consistent with our findings, in which the mean patient age was over 62 years and the DSS was estimated at 43%. Despite significant advancements in cancer therapy, particularly in the field of precision oncology, surgical resection remains the cornerstone of treatment for OSCC when feasible [23]. However, even with clear microscopic margins and the use of adjuvant radiotherapy—often combined with chemotherapy—achieving durable disease control remains a challenge. Recurrence rates in advanced-stage disease remain high, ranging from 30% to 40% [24]. These figures are consistent with our findings, in which the recurrence rate was approximately 29%. The typically late diagnosis of OSCC, often at T3 or T4 stage and frequently accompanied by nodal involvement, further complicates treatment and contributes to poor overall outcomes [25]. Therefore, an individualized treatment approach that incorporates both clinical and pathological patient-specific variables may enhance risk stratification and support the development of more effective, tailored treatment strategies

Thus, the aim of this study was to evaluate a range of clinical and pathological variables that may influence DSS in patients with OSCC. By identifying factors associated with poorer outcomes, we sought to improve risk stratification through the use of prognostic markers and to support the development of more personalized and effective treatment strategies.

The clinical variables assessed in our study—although well-established risk factors for carcinogenesis—did not demonstrate a statistically significant impact on DSS. Factors such as smoking history [26,27], which is frequently associated with comorbidities like diabetes mellitus and atherosclerosis [28,29], as well as alcohol use [30,31,32], are known to substantially increase the risk of developing oral cancer. Likewise, advanced age, which reflects prolonged exposure to carcinogenic factors, has been suggested as a potential contributor to poorer outcomes [33]. Despite the recognized role of postoperative radiotherapy RTH and CHTH in improving survival, our analysis did not reveal a statistically significant association between these treatment modalities and DSS in the studied cohort.

Nevertheless, further analysis of clinical and pathological variables revealed several noteworthy correlations. In particular, neuroinvasion, which showed a significant association with DSS in our cohort, is a well-established marker of aggressive tumor behavior [34]. It is commonly linked to higher rates of local recurrence, distant metastasis, and poor overall survival [34,35]. In a study conducted by Hui-Wen et al., perineural invasion remained an independent predictor of worse OS [36]. Tumor spread along nerve sheaths may enable cancer cells to extend beyond surgical margins, increasing the risk of incomplete resection and subsequent recurrence [37] as it is often associated with deeper tissue infiltration and more advanced T-stage tumors [38]. These characteristics underscore the prognostic significance of perineural invasion, aligning with our findings. Therefore, incorporating this feature into treatment planning may improve risk stratification and support the development of more personalized therapeutic strategies aimed at enhancing oncologic outcomes.

Another important correlation identified in our study was the association between angioinvasion and the administration of postoperative CHTH. Angioinvasion, characterized by the infiltration of cancer cells into blood vessels, is a well-established marker of tumor aggressiveness and is associated with an increased risk of hematogenous spread [39]. In patients with OSCC, its presence has been linked to a higher likelihood of distant metastasis and poorer prognosis [40]. In a study performed by Spoerl et al., there was decreased five-year OS and RFS (recurrence-free survival) values in patients with confirmed angioinvasion [41]. Consequently, the identification of angioinvasion should play a critical role in treatment planning, particularly in guiding decisions regarding the use of postoperative systemic therapy.

Among other pathological variables assessed in our study, lymphatic invasion demonstrated a significant correlation with increased tumor volume. This finding suggests that tumor volume may reflect not only the physical size of the tumor but also its underlying biological aggressiveness [42]. Additionally, we observed an inverse correlation between T1 tumors and lymphatic invasion, indicating that smaller tumors are less likely to infiltrate lymphatic vessels. These results support the hypothesis that tumor volume may serve as an indirect predictor of nodal metastasis or extranodal extension [43]. Consequently, tumor volume may represent a valuable preoperative imaging-based biomarker for identifying patients at elevated risk of lymphatic invasion. Incorporating this parameter into clinical decision-making could aid in tailoring surgical margins, selecting patients for elective neck dissection, and guiding adjuvant treatment strategies—even in cases where nodal involvement is not clinically apparent.

Moreover, lymphatic invasion was significantly correlated with both angioinvasion and neuroinvasion, suggesting a tendency for these invasive features to co-occur [44]. This association reflects a more aggressive tumor phenotype, where the cancer not only infiltrates lymphatic vessels but also demonstrates the capacity to invade blood vessels and nerve sheaths. Therefore, the concurrent presence of these invasion patterns may indicate enhanced tumor invasiveness and a higher likelihood of regional or distant spread.

In addition, angioinvasion was significantly correlated with higher tumor grades, specifically G2 and G3, indicating a strong association between vascular infiltration and poor differentiation. It has already been broadly studied that both poorly differentiated tumors (G3) and moderately differentiated tumors (G2) typically exhibit more aggressive biological behavior, including increased proliferative activity, loss of cellular cohesion, and enhanced invasive potential [19,45]. Therefore, less differentiated tumors are more likely to invade blood vessels, facilitating hematogenous spread, contributing to worse clinical outcomes. This relationship highlights the importance of tumor grading in predicting vascular invasion and reinforces the role of angioinvasion as a marker of tumor aggressiveness.

However, although multivariable Cox regression analysis did not find pN+ status or perineural invasion statistically significant for predicting time-to-event outcomes, both univariate and multivariate logistic regression analyses identified them as significant and independent predictors of DSS in patients with OSCC. These results reflect the established impact of nodal metastasis on survival outcomes [46,47], with Zanoni et al. reporting that confirmed lymph node metastases reduce survival rates to approximately 50% [48]. The differing significance of perineural invasion and pN+ status between logistic and Cox regression models likely reflects the impact of time-to-event considerations. While both were associated with disease-specific mortality in logistic regression, their effects may diminish over time, as captured by Cox analysis. Censoring and follow-up variability may also influence hazard estimates, highlighting the value of using complementary statistical approaches in prognostic assessment.

Kaplan–Meier analysis further demonstrated that angioinvasion significantly reduced survival, with neuroinvasion having an even greater impact. Notably, pN+ status emerged as the strongest predictor of poor survival. These findings underscore the independent contributions of perineural invasion and lymph node involvement to cancer-specific mortality and highlight the importance of incorporating detailed pathological features—such as perineural invasion, angioinvasion, and pN+ status [49,50]—into risk assessment frameworks to improve treatment planning for high-risk OSCC patients.

Additionally, our analysis confirmed that the simultaneous presence of perineural, angioinvasion, and lymphatic invasion significantly impacts patient prognosis. Patients exhibiting all three invasion types were markedly more likely to be deceased at follow-up (86%, 12/14) compared to those without this combination (49%, 42/86). Moreover, patients with concurrent perineural, vascular, and lymphatic invasion had over six times greater odds of death during follow-up than those without this constellation of high-risk features. These findings highlight the cumulative adverse effect of multiple invasive pathways and support the inclusion of such composite histopathological indicators in future prognostic models for oral cavity cancer.

Nevertheless, this study has several limitations that should be acknowledged. Its retrospective design may introduce selection bias and limit the ability to establish causal relationships. In addition, the sample size, while clinically relevant, may not provide sufficient statistical power to detect smaller effect sizes. Moreover, one key limitation of this study is the relatively short median follow-up duration of 12 months. While this timeframe allowed for the assessment of early oncologic outcomes, it may underestimate the true incidence of late recurrences or long-term survival trends. A follow-up period of at least 5 years is considered standard for oral cavity cancer, and future studies with extended follow-up will be important to validate and expand upon these findings. Furthermore, data were derived from a single institution, which may affect the generalizability of the findings to broader populations with differing demographic or clinical characteristics. However, despite these limitations, the study offers valuable insights into prognostic factors in OSCC and lays the groundwork for future prospective, multi-center research studies with a larger patient cohort.

This study underscores the prognostic significance of key pathological features—such as angioinvasion, perineural invasion, lymph node involvement (pN+), and tumor volume—in patients with OSCC. While TNM staging remains central to clinical decision-making, our findings emphasize the need for more refined risk-stratification tools that incorporate additional markers of tumor aggressiveness. Integrating these variables into routine pathological evaluation and treatment planning could improve prognostic accuracy and support more individualized therapeutic approaches for high-risk patients. A major strength of this study is its well-defined, homogenous patient cohort, all treated and followed within a single institution using consistent protocols. Comprehensive clinicopathological data were collected, including quantified lifestyle risk factors (smoking-years and alcohol-years), detailed tumor characteristics, and the presence of simultaneous angioinvasion, perineural invasion, and lymphatic invasion. The inclusion of actual postoperative treatment dose data for RTH and CHTH further enhances the clinical applicability of the results. Notably, the study demonstrates the cumulative negative impact of multiple invasive features, reinforcing their potential role in future prognostic models. These findings merit validation in larger, prospective, and multicenter cohorts. Future research should also explore the integration of composite histopathological markers with molecular and genomic data to enhance prognostic precision and guide more personalized treatment strategies for patients with high-risk OSCC.

## 5. Conclusions

This study reinforces the prognostic relevance of key histopathological features—particularly neuroinvasion, angioinvasion, lymphatic invasion, and nodal metastasis—in patients with OSCC. While TNM staging remains an essential tool in clinical oncology, our findings demonstrate that it alone is insufficient to fully capture the biological behavior and clinical trajectory of OSCC. The incorporation of detailed invasion markers and tumor volume into prognostic models significantly enhances risk stratification and may support more precise, personalized treatment decisions. These insights advocate for a multidimensional approach to OSCC management, in which both anatomical staging and biological indicators of tumor aggressiveness guide therapeutic planning. Future prospective studies with larger, multi-institutional cohorts are warranted to validate these findings and to develop refined prognostic frameworks that integrate histopathology, clinical data, and molecular profiling.

## Figures and Tables

**Table 1:** Patient characteristics.

Variable	*N* (%)
Age	62.09 ± 10.28 (M ± SD)
Gender M	57 (57.0%)
F	43 (43.0%)
DM 2	15 (15%)
Smoking	60 (60%)
Smoking-years	25.73 ± 9.65 1(M ± SD)
Alcohol use	45 (45%)
Alcohol-years	8.72 ± 4.79 (M ± SD)
Peripheral atherosclerosis	40 (40%)
RTH post-surgery	64 (64%)
RTH mean dose	62.15 ± 2.90 (M ± SD)
CHTH post-surgery	8 (8%)
CHTH mean dose	244.44 ± 101.38 (M ± SD)
Overall Survival	46 (46 %)
DSS	43 (43.0%)
Recurrence	29 (29%)
Local recurrence	23 (79.3%)
Regional recurrence	6 (20.7%)
Recurrence time (months)	14.4 ± 11.9 (M ± SD)
ECOG 0	33 (33%)
ECOG 1	67 (67%)

**Table 2:** Tumor characteristics.

Variable	*N* (%)
Tumor histology	
SCC	100 (100%)
Tumor volume in cm3	34.55 ± 59.38 (M ± SD)
Angioinvasion	28 (28%)
Lymphatic invasion	42 (42%)
Perineural invasion	44 (44%)
pENE	23 (23%)
pN+	37 (37%)
Grading	
G1	8 (8%)
G2	68 (64%)
G3	24 (24%)
TNM	
T1	6 (6%)
T2	25 (25%)
T3	27 (27%)
T4	42 (42%)
N0	54 (54%)
N1	19 (19%)
N2	25 (25%)
N3	2 (2%)

**Table 3:** Clinicopathologic features by tumor location.

	*N* (%)	Age (Mean ± SD)	Male (%)	Female (%)	Smoking Years (Mean ± SD)	Alcohol Years (Mean ± SD)	RTH (%)	CHTH (%)	T1 (%)	T2 (%)	T3 (%)	T4 (%)
Gum	11 (11%)	62.9 ± 6.9	7 (64%)	4 (36%)	30.6 ± 8.1	6.8 ± 2.6	8 (73%)	0 (0%)	0 (0%)	2 (18%)	1 (9%)	8 (73%)
Tongue	38 (38%)	59.9 ± 10.5	24 (63%)	14 (37%)	27.9 ± 8.4	9.7 ± 5.5	21 (55%)	3 (8%)	2 (5%)	15 (39%)	10 (26%)	9 (24%)
Floor of the mouth	29 (29%)	62.1 ± 9.7	13 (45%)	16 (55%)	21.7 ± 11.3	8.6 ± 4.9	23 (79%)	4 (14%)	2 (7%)	5 (17%)	6 (21%)	15 (52%)
Hard palate	2 (2%)	47.0 ± 32.5	2 (100%)	0 (0%)	nan ± nan	15.0 ± nan	0 (0%)	0 (0%)	1 (50%)	0 (0%)	0 (0%)	1 (50%)
Cheek mucosa	13 (13%)	68.1 ± 7.9	6 (46%)	7 (54%)	22.5 ± 8.7	5.8 ± 2.0	9 (69%)	1 (8%)	1 (8%)	3 (23%)	5 (38%)	4 (31%)
Lower lip	6 (6%)	63.3 ± 3.4	5 (83%)	1 (17%)	21.7 ± 7.6	15.0 ± nan	3 (50%)	0 (0%)	0 (0%)	0 (0%)	2 (33%)	4 (67%)
Upper lip	1 (1%)	80.0 ± nan	0 (0%)	1 (100%)	30.0 ± nan	8.0 ± nan	1 (100%)	0 (0%)	0 (0%)	0 (0%)	1 (100%)	0 (0%)

**Table 4:** Clinical variables evaluation.

Clinical Variable	Perineural Invasion	Angioinvasion	Lymphatic
Age (mean)	−0.057	0.077	0.096
Gender	0.037	0.002	0.043
Smoking	−0.099	−0.082	0.033
Smoking-years	−0.045	0.214	0.141
Alcohol	−0.032	0.152	0.086
Alcohol-years	−0.245	0.041	−0.099
DM 2	−0.09	−0.012	−0.017
Peripheral atherosclerosis	0.063	0.011	−0.104
post-RTH	0.059	−0.009	0.072
post-RTH mean dose	0.127	−0.132	**0.684**
post-CHTH	0.11	**0.309**	0.048
post-CHTH mean dose	−0.164	0.329	0.286
Death from disease	**0.403**	0.134	0.081
Disease recurrence	0.188	0.141	0.081

Values represent Pearson correlation coefficients. Bolded values are statistically significant (*p* < 0.05). Italicized values represent moderate correlations (|*r*| ≥ 0.3) that were not statistically significant. Abbreviations: RTH = radiotherapy; CHTH = chemotherapy; DM2 = type 2 diabetes mellitus.

**Table 5:** Correlation between key pathological features and invasion markers (angioinvasion, neuroinvasion, and lymphatic invasion.

Pathological Variable	Neuroinvasion	Angioinvasion	Lymphatic Invasion
Tumor volume	0.0697	−0.0809	**0.3992**
T1	−0.0543	−0.0638	**−0.215**
T2	−0.0202	−0.1665	−0.0887
T3	−0.0853	0.1224	−0.0155
T4	−0.0378	0.0624	0.1686
pN+	**0.238**	0.4625	0.193
G1	−0.1129	−0.1839	0.1225
G2	−0.0397	**−0.2406**	−0.0243
G3	0.1151	**0.3796**	−0.0512
pENE	0.0536	−0.1414	**0.5186**
Gum	−0.077	−0.077	−0.077
Tongue	0.062	0.062	0.062
Floor of the mouth	−0.006	−0.006	−0.006
Hard palate	0.07	0.07	0.07
Cheek mucosa	−0.042	−0.042	−0.042
Lower lip	0.03	0.03	0.03
Upper lip	−0.063	−0.063	−0.063
Angioinvasion	**0.3412**		**0.2809**
Neuroinvasion		**0.3412**	**0.2159**
Lymphatic invasion	**0.2159**	**0.2809**	

Values represent Pearson correlation coefficients. Bolded values are statistically significant (*p* < 0.05). Italicized values represent moderate correlations (|*r*| ≥ 0.3) that were not statistically significant. Abbreviations: pENE = pathological extranodal extension.

**Table 6:** Univariate logistic regression.

**Variable**	**Odds Ratio**	**CI Lower**	**CI Upper**	***p*-Value**
pN+	3.841	1.61	9.161	0.0024
Angioinvasion	1.826	0.751	4.443	0.1841
Perineural invasion	5.63	2.362	13.42	0.0001
Lymphatic invasion	1.39	0.626	3.084	0.4183
Tumor volume in cm3	1.002	0.996	1.009	0.4839
pENE	1.405	0.55	3.59	0.4769

Abbreviations: CI—confidence interval.

**Table 7:** Multivariate logistic regression.

	Odds Ratio	CI Lower	CI Upper	*p*-Value
pN+	4.837	1.974	11.852	0.0006
Perineural invasion	3.079	1.215	7.804	0.0178

Abbreviations: CI—confidence interval.

**Table 8:** Cox regression.

Variable	Category 0 (*n*, %)	Category 1 (*n*, %)	HR (Univariable)	HR (Multivariable)
pN+	25 (46.3%)	29 (53.7%)	1.69 (0.88–3.24), *p* = 0.117	1.68 (0.88–3.24), *p* = 0.118
Perineural invasion	20 (37.0%)	34 (63.0%)	1.05 (0.54–2.08), *p* = 0.877	1.02 (0.52–2.02), *p* = 0.944

**Table 9:** Triple invasion subgroup analysis.

Variable	Value
Gender	
Male	11 (78.6%)
Female	3 (21.4%)
Age	
Mean ± SD	59.6 ± 10.3 years
Tumor volume	
Mean ± SD	37.8 ± 20.5 cm^3^
Reccurence	
Yes	9 (64.3%)
No	5 (35.7%)
Death	
Deceased	10 (71.4%)
Statistic	Value
Chi-square	5.191
*p*-value	0.0227
Degrees of Freedom	1.0
Logistic Regression	Patients with all three invasions
Coefficient	1.838
Odds Ratio	6.285
CI Lower	1.326
CI Upper	29.778
*p*-value	0.020

Abbreviations: CI—confidence interval.

## Data Availability

All data supporting the findings of this study are available upon reasonable request from the corresponding author.

## Abbreviations

The following abbreviations are used in this manuscript:

AJCC/CAP	American Joint Committee on Cancer/College of American Pathologists
CHTH	Chemotherapy
CI	Confidence Interval
DOD	Death of Disease
DOI	Depth of Invasion
DM	Diabetes Mellitus
DSS	Disease-Specific Survival
ECOG	Eastern Cooperative Oncology Group
H&E	Hematoxylin & Eosin
HPV	Human Papillomavirus
HR	Hazard Ratio
IARC	International Agency for Research on Cancer
IHC	Immunohistochemistry
IRB	Institutional Review Board
IMRT	Intensity-Modulated Radiation Therapy
M	Mean
NCCN	National Comprehensive Cancer Network
OR	Odds Ratio
OS	Overall Survival
OSCC	Oral Squamous Cell Carcinoma
pENE	Pathological Extranodal Expression
PORT	Postoperative Radiotherapy
RFS	Recurrence Free Survival
RTH	Radiotherapy
SCC	Squamous Cell Carcinoma
SD	Standard Deviation BMC Cancer 2024

## References

[1] Sung H., Ferlay J., Siegel R.L., Laversanne M., Soerjomataram I., Jemal A., Bray F. (2021). Global Cancer Statistics 2020: GLOBOCAN Estimates of Incidence and Mortality Worldwide for 36 Cancers in 185 Countries. CA A Cancer J. Clin..

[2] International Agency for Research on Cancer (2023). IARC Handbooks of Cancer Prevention, Volume 19: Oral Cancer Prevention.

[3] Fridman E., Na’ARa S., Agarwal J., Amit M., Bachar G., Villaret A.B., Brandao J., Cernea C.R., Chaturvedi P., Clark J. (2018). The role of adjuvant treatment in early-stage oral cavity squamous cell carcinoma: An international collaborative study. Cancer.

[4] Alim N., Elsheikh M., Satti A.A., Tabassum N., Suleiman A.M. (2024). Recurrence of oral squamous cell carcinoma in surgically treated patients at Khartoum Teaching Dental Hospital retrospective cross-sectional study. BMC Cancer.

[5] Jadhav K., Gupta N., Kb J. (2013). Clinicopathological prognostic implicators of oral squamous cell carcinoma: Need to understand and revise. North Am. J. Med Sci..

[6] Lindenblatt R.d.C.R., Martinez G.L., Silva L.E., Faria P.S., Camisasca D.R., Lourenço S.d.Q.C. (2011). Oral squamous cell carcinoma grading systems–analysis of the best survival predictor. J. Oral Pathol. Med..

[7] Castellsagué X., Alemany L., Quer M., Halec G., Quirós B., Tous S., Clavero O., Alòs L., Biegner T., Szafarowski T. (2016). HPV Involvement in Head and Neck Cancers: Comprehensive Assessment of Biomarkers in 3680 Patients. J. Natl. Cancer Inst..

[8] Jayaprakash V., Reid M., Hatton E., Merzianu M., Rigual N., Marshall J., Gill S., Frustino J., Wilding G., Loree T. (2011). Human papillomavirus types 16 and 18 in epithelial dysplasia of oral cavity and oropharynx: A meta-analysis, 1985–2010. Oral Oncol..

[9] Singh D., Pandey M., Dhiman V.K., Sharma A., Pandey H., Verma S.K., Pandey R. (2024). Personalized medicine: An alternative for cancer treatment. Cancer Treat. Res. Commun..

[10] Schmidt K.T., Chau C.H., Price D.K., Figg W.D. (2016). Precision Oncology Medicine: The Clinical Relevance of Patient-Specific Biomarkers Used to Optimize Cancer Treatment. J. Clin. Pharmacol..

[11] Ghantous Y., Nashef A., Sidransky D., Abdelraziq M., Alkeesh K., Araidy S., Koch W., Brait M., Abu El-Naaj I. (2022). Clinical and Prognostic Significance of the Eighth Edition Oral Cancer Staging System. Cancers.

[12] National Comprehensive Cancer Network Head and Neck Cancer (Version 2.2025). https://www.nccn.org/professionals/physician_gls/pdf/head-and-neck.pdf.

[13] Rizzo A., Mollica V., Cimadamore A., Santoni M., Scarpelli M., Schiavina R., Cheng L., Lopez-Beltran A., Brunocilla E., Montironi R. (2021). TNM staging towards a personalized approach in metastatic urothelial carcinoma: What will the future be like?—A narrative review. Transl. Androl. Urol..

[14] Giacomelli L., Sacco R., Papa S., Carr B.I. (2023). Understanding the Drawbacks of the Current Tumor Staging Systems: How to Improve?. Cancers.

[15] Holthoff E.R.B., Jeffus S.K., Gehlot A., Stone R., Erickson S.W., Kelly T., Quick C.M., Post S.R. (2015). Perineural Invasion Is an Independent Pathologic Indicator of Recurrence in Vulvar Squamous Cell Carcinoma. Am. J. Surg. Pathol..

[16] Russo D., Mariani P., Caponio V.C.A., Russo L.L., Fiorillo L., Zhurakivska K., Muzio L.L., Laino L., Troiano G. (2021). Development and Validation of Prognostic Models for Oral Squamous Cell Carcinoma: A Systematic Review and Appraisal of the Literature. Cancers.

[17] Saidak Z., Piazza C. (2022). Editorial: Oral Oncology: From Precise Surgery to Precision Medicine and Surgery. Front. Oral Heal..

[18] Liebig C., Ayala G., Wilks J.A., Berger D.H., Albo D. (2009). Perineural invasion in cancer. Cancer.

[19] Tan Y., Wang Z., Xu M., Li B., Huang Z., Qin S., Nice E.C., Tang J., Huang C. (2023). Oral squamous cell carcinomas: State of the field and emerging directions. Int. J. Oral Sci..

[20] Al-Hakami H.A., Al-Talhi A.A., AlRajhi B., Alshareef M.A., Awad B.I., Hussain T., Al-Garni M. (2025). Oncological outcomes, survival analysis, and failure patterns in patients with resectable squamous cell carcinoma of the oral tongue treated with glossectomy. Egypt. J. Otolaryngol..

[21] Tranby E.P., Heaton L.J., Tomar S.L., Kelly A.L., Fager G.L., Backley M., Frantsve-Hawley J. (2022). Oral Cancer Prevalence, Mortality, and Costs in Medicaid and Commercial Insurance Claims Data. Cancer Epidemiol. Biomark. Prev..

[22] Safi A.-F., Kauke M., Grandoch A., Nickenig H.-J., Zöller J.E., Kreppel M. (2017). Analysis of clinicopathological risk factors for locoregional recurrence of oral squamous cell carcinoma–Retrospective analysis of 517 patients. J. Cranio-Maxillofac. Surg..

[23] Mohamad I., Glaun M.D., Prabhash K., Busheri A., Lai S.Y., Noronha V., Hosni A. (2023). Current Treatment Strategies and Risk Stratification for Oral Carcinoma. Am. Soc. Clin. Oncol. Educ. Book.

[24] Al-Sarraf M. (2002). Treatment of Locally Advanced Head and Neck Cancer: Historical and Critical Review. Cancer Control..

[25] Swaminathan D., George N.A., Thomas S., Iype E.M. (2024). Factors associated with delay in diagnosis of oral cancers. Cancer Treat. Res. Commun..

[26] Eloranta R., Vilén S.-T., Keinänen A., Salo T., Qannam A., Bello I.O., Snäll J. (2024). Oral squamous cell carcinoma: Effect of tobacco and alcohol on cancer location. Tob. Induc. Dis..

[27] Colares N., Rodrigues D.F.S., Freitas M.O., Dantas T.S., Cunha M.D.P.S.S., Sousa F.B., Silva P.G.d.B. (2019). Smoking History Decreases Survival in Patients with Squamous Cell Carcinoma of the Mouth: A Retrospective Study with 15 Years of Follow-up. Asian Pac. J. Cancer Prev..

[28] Katsi V., Papakonstantinou I., Tsioufis K. (2023). Atherosclerosis, Diabetes Mellitus, and Cancer: Common Epidemiology, Shared Mechanisms, and Future Management. Int. J. Mol. Sci..

[29] Remschmidt B., Pau M., Gaessler J., Zemann W., Jakse N., Payer M., Végh D. (2022). Diabetes Mellitus and Oral Cancer: A Retrospective Study from Austria. Anticancer. Res..

[30] Ogden G.R. (2018). Alcohol and mouth cancer. Br. Dent. J..

[31] Ferraguti G., Terracina S., Petrella C., Greco A., Minni A., Lucarelli M., Agostinelli E., Ralli M., de Vincentiis M., Raponi G. (2022). Alcohol and Head and Neck Cancer: Updates on the Role of Oxidative Stress, Genetic, Epigenetics, Oral Microbiota, Antioxidants, and Alkylating Agents. Antioxidants.

[32] Mores A.L., Bonfim-Alves C.G., López R.V.M., Rodrigues-Oliveira L., Palmier N.R., Mariz B.A.L.A., Migliorati C.A., Kowalski L.P., Santos-Silva A.R., Brandão T.B. (2024). Prognostic Factors in Head and Neck Cancer: A Retrospective Cohort Study of 3052 Patients in Brazil. Oral Dis..

[33] Badwelan M., Muaddi H., Ahmed A., Lee K.T., Tran S.D. (2023). Oral Squamous Cell Carcinoma and Concomitant Primary Tumors, What Do We Know? A Review of the Literature. Curr. Oncol..

[34] Mishra A., Das A., Dhal I., Shankar R., Bhavya B., Singh N., Tripathi P., Daga D., Rai A., Gupta M. (2022). Worst pattern of invasion in oral squamous cell carcinoma is an independent prognostic factor. J. Oral Biol. Craniofacial Res..

[35] Quintana D.M.V.O., Dedivitis R.A., Kowalski L.P. (2022). Prognostic impact of perineural invasion in oral cancer: A systematic review. Acta Otorhinolaryngol. Ital..

[36] Cheng H.-W., Lin L.-H., Lin H.-P., Liu C.-J. (2025). Perineural Invasion Unveiled: Deciphering the Prognostic Impact of Diameter and Quantity Subcategories in Oral Cancer. J. Otolaryngol.–Head Neck Surg..

[37] Misztal C.I., Green C., Mei C., Bhatia R., Torres J.M.V., Kamrava B., Moon S., Nicolli E., Weed D., Sargi Z. (2021). Molecular and Cellular Mechanisms of Perineural Invasion in Oral Squamous Cell Carcinoma: Potential Targets for Therapeutic Intervention. Cancers.

[38] Cuéllar I.N., Alonso S.E., Serrano F.A., Herrera I.H., León J.J.Z., Vera J.L.D.C.P.d., López A.M.L., Muela C.M., de Frutos G.A., Caicoya S.O. (2023). Depth of Invasion: Influence of the Latest TNM Classification on the Prognosis of Clinical Early Stages of Oral Tongue Squamous Cell Carcinoma and Its Association with Other Histological Risk Factors. Cancers.

[39] Adel M., Kao H.-K., Hsu C.-L., Huang J.-J., Lee L.-Y., Huang Y., Browne T., Tsang N.-M., Chang Y.-L., Chang K.-P. (2015). Evaluation of Lymphatic and Vascular Invasion in Relation to Clinicopathological Factors and Treatment Outcome in Oral Cavity Squamous Cell Carcinoma. Medicine.

[40] Ting K.-C., Lee T.-L., Li W.-Y., Chang C.-F., Chu P.-Y., Wang Y.-F., Tai S.-K. (2021). Perineural invasion/lymphovascular invasion double positive predicts distant metastasis and poor survival in T3–4 oral squamous cell carcinoma. Sci. Rep..

[41] Spoerl S., Gerken M., Fischer R., Mamilos A., Spoerl S., Wolf S., Pohl F., Klingelhöffer C., Ettl T., Reichert T.E. (2020). Lymphatic and vascular invasion in oral squamous cell carcinoma: Implications for recurrence and survival in a population-based cohort study. Oral Oncol..

[42] Mijatov I., Kiralj A., Ilić M.P., Vučković N., Spasić A., Nikolić J., Tadić A., Mijatov S. (2023). Pathological tumor volume as a simple quantitative predictive factor of survival in oral squamous cell carcinoma. Oncol. Lett..

[43] Lucchi E., Cercenelli L., Maiolo V., Bortolani B., Marcelli E., Tarsitano A. (2023). Pretreatment Tumor Volume and Tumor Sphericity as Prognostic Factors in Patients with Oral Cavity Squamous Cell Carcinoma: A Prospective Clinical Study in 95 Patients. J. Pers. Med..

[44] Viswanatha S.C., Hedne N., Hasan S. (2018). Correlation between histological grading, LVI and PNI of carcinoma oral tongue to lymph node metastasis. Int. J. Otorhinolaryngol. Head Neck Surg..

[45] Williams H.K. (2000). Molecular pathogenesis of oral squamous carcinoma. Mol. Pathol..

[46] Haidari S., Obermeier K.T., Kraus M., Otto S., Probst F.A., Liokatis P. (2022). Nodal Disease and Survival in Oral Cancer: Is Occult Metastasis a Burden Factor Compared to Preoperatively Nodal Positive Neck?. Cancers.

[47] Das K., Gontu G.S.S.R., Aasumi K., Das R.J., Das A., Rahman T., Das A.K., Kakati K. (2024). Occult Metastasis: Incidence, Pattern, and Impact on Survival in Patients with Oral Cancer, pN0 vs pN1 in a Cohort of cN0. A Prospective Cohort Study. Indian J. Otolaryngol. Head Neck Surg..

[48] Ferlito A., Rinaldo A., Devaney K.O., MacLennan K., Myers J.N., Petruzzelli G.J., Shaha A.R., Genden E.M., Johnson J.T., de Carvalho M.B. (2002). Prognostic significance of microscopic and macroscopic extracapsular spread from metastatic tumor in the cervical lymph nodes. Oral Oncol..

[49] Tsai M.-H., Lin Y.-T., Chuang H.-C., Huang T.-L., Lu H., Chien C.-Y., Fang F.-M. (2022). Prognostic Value of Pathologically Positive Nodal Number in p16-Negative Oropharyngeal and Hypopharyngeal Squamous Cell Carcinoma with pN3b Status. Diagnostics.

[50] Cheng C.-S., Chen C.-C., Liu Y.-C., Wang C.-C., Chou Y.-S. (2022). Peri-Neural Invasion Is an Important Prognostic Factor of T2N0 Oral Cancer. Medicina.

